# Effects and Adaptive Responses of Sulfate-Reducing Biochemical System to Acid Stress

**DOI:** 10.3390/biom16030444

**Published:** 2026-03-16

**Authors:** Yanmei Zhang, Bei Zhao, Jiang Li, Tao Yuan, Yajie Liu, Zhanxue Sun

**Affiliations:** 1 Jiangxi Provincial Key Laboratory of Genesis and Remediation of Groundwater Pollution, East China University of Technology, Nanchang 330013, China; zhangym@ecut.edu.cn (Y.Z.); 199560037@ecut.edu.cn (Y.L.);; 2 State Key Laboratory of Nuclear Resources and Environment, East China University of Technology, Nanchang 330013, China; 201960043@ecut.edu.cn; 3 School of Water Resources and Environmental Engineering, East China University of Technology, Nanchang 330013, China; 4 School of Water Resources and Environment, China University of Geosciences (Beijing), Beijing 100083, China; bzhao@cugb.edu.cn; 5 Normal College, East China University of Technology, Nanchang 330013, China

**Keywords:** acid stress, biological sulfate reduction, physiological response, microbial community, functional gene, acid-adaptive mechanism

## Abstract

A decrease in pH can affect the biochemical properties of a sulfate reduction system, but the stress responses to such pH fluctuations and acid-adaptive mechanisms of the microorganisms remain incompletely understood. Here, we compared the sulfate (SO_4_^2−^) reduction performance of a sulfate-reducing consortium (SRB system) and a pure *Desulfovibrio* sp. system (Des. system, control) under pH 7.0, 5.5, and 5.0 via batch experiments. A key novelty is the integration of microbial physiology and metagenomics to reveal adaptive mechanisms: the Des. system showed significant inhibition of growth and sulfate reduction with decreasing pH, while the SRB system maintained superior SO_4_^2−^ removal efficiency through three synergistic adjustments: (1) physiological regulation (enhanced H^+^-ATPase activity, stress protein production, and cell membrane cyclopropane fatty acid content); (2) microbial community restructuring (enrichment of acid-resistant *Bacillus* and *Clostridium*); and (3) functional gene upregulation (sulfate import, dissimilar sulfate reduction, sulfide oxidation, and SO_x_ system-related genes, *p* < 0.05). This study links physiological responses to metagenomic functional shifts under acid stress, providing critical theoretical support for applying sulfate-reducing consortia in acidic sulfate-containing wastewater remediation.

## 1. Introduction

The discharge of wastewater containing a high amount of sulfates generated from industrial activities poses a threat to freshwater environments [1]. A variety of strategies have been deployed for the remediation of such sulfate-rich wastewater, including chemical precipitation [2,3], ion exchange, adsorption [4,5], bioremediation [6], etc. Among them, bioremediation has been considered to be one of the most promising strategies due to its high remediation efficiency, low cost, and environmental friendliness [7]. Sulfate-reducing bacteria (SRB) are generally regarded as the most dominant species for remediation of sulfate pollution, oxidizing reducing agents such as organic compounds or H2 while using sulfate as a final electron acceptor and producing sulfides [8].

*Desulfovibrio* sp. is a common sulfate-reducing bacterium in nature and is easy to culture in laboratory. *Desulfovibrio* is one of the most prevalent and extensively studied SRB in nature. Its metabolic mechanisms—such as utilizing sulfate as the final electron acceptor and oxidizing organic matter or H_2_ to produce sulfide—closely align with the core characteristics of sulfate reduction. As a result, it serves as a model strain for elucidating the sulfate-reducing capabilities of individual SRB species. Most of studies have shown that *Desulfovibrio* sp. is viable in treating sulfate-containing wastewater [9,10]. However, the best treatment efficiency is usually obtained under the optimal growth conditions of *Desulfovibrio* sp., and the efficiency is easily affected by sudden changes in environmental conditions, especially pH [11,12]. This suggests that the poor stability of microbial systems composed of a single species is often an important limitation to the remediation efficiency in practical applications. Microbial communities combining other specific species can provide an opportunity to overcome this weakness. Studies have shown that the sulfate-reducing consortium can enhance the adaptability of sulfate reduction systems to adverse conditions and improve the stability of biochemical systems [13,14,15], which is of great significance in the practical remediation of sulfate wastewater. Numerous studies have confirmed the potential of *Desulfovibrio* for application in sulfate-containing wastewater treatment. However, its sulfate reduction efficiency is highly dependent on its optimal growth condition of neutral to slightly alkaline pH (7.0–7.5). Its instability in acidic environments has become a major bottleneck for practical applications. Selecting this strain as a control allows for a direct comparison of the performance differences between a single species and a composite microbial community under acid stress, thereby highlighting the advantages of the latter.

As one of the most important environmental factors, pH exerts a critical influence on the efficiency and stability of sulfate reduction systems. The optimal growth and treatment conditions of general SRB tend to be neutral conditions, such as pH 7.0–7.5, at which the SRB are more efficient at removing SO_4_^2−^ by reducing sulfate. However, the rate of sulfate reduction is usually inhibited with the decrease in pH [16,17]. In fact, weak-acid conditions are not rare for actual sulfate wastewater treatment, which may derive from wastewater itself or the addition of carbon sources into the manual treatment system [18,19]. Notably, the selected pH levels (5.5 and 5.0) in this study are consistent with the pH ranges of typical acidic sulfate-containing wastewater encountered in practical applications. For instance, acid mine drainage, one of the most representative high-sulfate acidic wastewaters, often has a pH range of 2.0–6.0 due to the oxidation of sulfide minerals (e.g., pyrite). Among them, pH 5.5 represents the typical weak-acid operating condition for many sulfate-reducing bioreactors treating AMD, as moderate acidity (pH 5.0–6.0) is common after preliminary neutralization (e.g., with limestone) to reduce extreme toxicity. In contrast, pH 5.0 reflects the near-extreme acidic conditions in practical scenarios, such as untreated or poorly neutralized AMD (pH 4.5–5.5) from coal or metal mines, or sulfate-rich wastewater from chemical processes (e.g., fermentation, electroplating) that generates acidic metabolites. These pH values are not arbitrarily set but are derived from extensive field monitoring data and engineering practice reports, ensuring that the study results can directly inform the design and operation of bioremediation systems for real acidic sulfate-containing wastewater.

Lots of scholars have studied the operation of sulfate reduction systems at lower pH and investigated the key factors affecting the performance of the biochemical reactors, including but not limited to the type of carbon sources [20,21,22], microbial inoculum [23,24], coexisting metal ions [25,26,27,28] or pollutants [29], etc. At the same time, a number of experiments have also been conducted to promote sulfate reduction and improve the effect of microbial remediation systems through various optimization methods, involving the modification of external amendments [30,31,32,33,34], optimization source habitat selection of SRB [35,36], development of novel biochemical system [37,38,39], adjustment of start-up mode [40], addition of pretreatments, etc. In these studies, the authors focused on the treatment performances and evaluated the validity of bioremediation systems by measuring physicochemical indicators, while also paying attention to the changes in community structure under acid stress. Only a few studies studied the expression of relevant functional genes [41], and research on physiological responses of microorganisms in sulfate-reducing remediation systems has not been reported so far. However, in some papers related to other fields, such as those studying the responses of other bacterial communities to acid stress [42,43] or the sulfate-reducing consortium confronting different kinds of environmental stresses (temperature [44,45], heavy metals [46], pollutants [47,48], etc.), it is essential to analyze from the perspective of physiology and/or metagenomics, which are important components of the studies on the operation and evaluation of biochemical systems under stress conditions. In other words, there are scarce comprehensive and in-depth insights about the sulfate reduction system under acid stress in terms of microbial adaption, including physiological responses, community evolution, functional gene expression, etc. Further studies should be carried out to elucidate the acid-adaptative mechanisms of the SRB system.

Based on existing research gaps (the sensitivity of single SRB strains to acid stress and the unclear acid adaptation mechanisms of composite microbial communities), this study proposes the following core hypotheses. (1) Acidic conditions (pH ≤ 5.5) significantly inhibit the growth and sulfate reduction function of pure *Desulfovibrio* cultures, with the inhibition mechanisms primarily related to disruption of cell membrane integrity and disturbances in energy metabolism. (2) Sulfate-reducing composite microbial communities (SRB systems) can alleviate the inhibitory effects of acid stress through multiple adaptation strategies (such as the enrichment of acid-tolerant strains, differential expression of functional genes, adjustment of cell membrane components, and enhanced H^+^-ATPase activity), thereby maintaining higher sulfate reduction efficiency. (3) The synergistic interaction between acid-tolerant bacteria (e.g., *Bacillus*, *Clostridium*) and functional bacteria (e.g., *Desulfovibrio*) within the composite microbial community, along with the enhanced expression of functional genes related to sulfate transport, reduction, and sulfide oxidation, constitutes the key mechanism underlying their superior acid adaptation capability compared to single strains.

The objectives of this study are to: (1) elucidate the impacts of pH decline on microbial growth in *Desulfovibrio* sp. and sulfate reduction performance in both the *Desulfovibrio* sp. (Des.) monoculture system and SRB system and (2) uncover the stress responses of sulfate-reducing consortia to acidic conditions and delineate the underlying acid-adaptive mechanisms of the SRB system. A comprehensive understanding of the acid-adaptive strategies of SRB consortia is critical for enhancing the sulfate removal efficiency and operational stability of bioremediation systems in practical applications.

## 2. Materials and Methods

### 2.1. Culture of Microorganisms

An artificially cultured *Desulfovibrio* sp. colony was obtained [49], whose sulfate-reducing ability reached 0.205 kg SO_4_^2−^/(m^3^·d) under neutral condition. A sulfate-reducing consortium from different natural habitats was also constructed [50]. Modified Postgate’s medium B was selected as the activation culture of the above strains, consisting of KH_2_PO_4_ (0.5 g/L), NH_4_Cl (1.0 g/L), CaCl_2_·6H_2_O (0.1 g/L), MgSO_4_·7H_2_O (2.0 g/L), Na_2_SO_4_ (1.0 g/L), yeast extract (0.5 g/L), and sodium lactate (2.0 g/L). The medium was adjusted to a pH of 7.0 ± 0.1 and sterilized at 121 °C for 20 min before inoculation of *Desulfovibrio* sp. and the sulfate-reducing consortium. Bacterial suspension was used for experiments in this paper when the optical density of culture reached above 0.8 after about 3 days at optimal temperature (30 °C).

### 2.2. Batch Experiments

The acid stress effect batch experiments on sulfate reduction were performed in 500 mL glass serum bottles, including two experiments: steady-state inhibition and recovery experiments. All the batch experiments were conducted in triplicate.

#### 2.2.1. Acid Stress Exposure Experiments

In this steady-state inhibition experiment, three pH gradients were established: the neutral control group (pH 7.0 ± 0.1), the moderate acid stress group (pH 5.5 ± 0.1, T1), and the strong acid stress group (pH 5.0 ± 0.1, T2). Each group included two parallel systems, the Des. system and the SRB system, with three replicates per group. The pH of the medium was adjusted to 7.0 ± 0.1 (control group), 5.5 ± 0.1 (acid stress-T1), and 5.0 ± 0.1 (acid stress-T2) by 1 mol/L HCl, respectively. Bacterial inoculum was harvested from incubation reactors by centrifugation at 6000 rpm and 4 °C for 10 min. The cell pellets were washed twice with sterilized saline (0.85 wt%) and centrifuged again under similar conditions. The cell pellets obtained were aseptically dispersed into serum bottles containing 400 mL of fresh sterilized medium to achieve an inoculation volume of 2.5 × 10^7^ cells/mL. Nitrogen gas was purged into the reactors to maintain anaerobic conditions, and then the steady-state inhibition experiments were carried out in an incubator at 30 °C for nearly 15 days. Samples were collected at regular intervals using sterile syringes for analysis. The experimental groups of the pH-influencing study are shown in Table S1.

#### 2.2.2. Recovery Experiments

After the steady-state inhibition experiments above, the SO_4_^2−^ concentration and OD_600_ in Des.-T2 and SRBs-T2 were measured again on the next day by restoring pH to normal pH levels, namely 7.0 without acid stress. Recoverability of the Des. and SRB systems was evaluated by comparing the indicators measured from the steady-state inhibition experiments and the recovery experiments [51].

### 2.3. Analytical Methods

Physicochemical indicators and microbial growth characteristics were determined. pH was measured using an acidometer (ST3100, Ohaus Instrument (Changzhou) Co., Ltd., Changzhou, Jiangsu, China) and OD_600_ was analyzed with a UV–visible spectrophotometer (UV-1601, Shimadzu (China) Co., Ltd., Beijing, China). The concentration of SO_4_^2−^ was measured by ion chromatograph (CIC-D160, ShengHan Instrument Co., Ltd., Qingdao, Shandong, China) equipped with an SH-AC-11 column after samples were filtered through 0.45 µm filters. The number of bacteria was counted using the most probable number (MPN) method based on the examination of bacteria and algae in industrial circulating cooling water (GB/T 14643.5-2009 [52]). The percent cell survival at lower pH relative to that at pH 7.0 was calculated by the dilution plate counting method [53].

Microbial physiological response indicators were detected as follows. The ATP hydrolase (H^+^-ATPase) activity and the intracellular ATP concentration were determined using the H^+^-ATPase assay kit (GA069-1, Nanjing Jiancheng Bioengineering Research Institute, Nanjing, China) and ATP content assay kit (A095-1-1, Nanjing Jiancheng Bioengineering Research Institute, Nanjing, China), respectively, both following the manufacturers’ protocols. The content of intracellular protein (PN) was measured following the diquinolinic acid (BCA) procedure [54]. The capsular polysaccharide (PS) was detected using the phenol-sulfuric method. The membrane permeability was determined according to related literature [55]. Extraction of fatty acids in the cell membrane was carried out based on previous reports [56] and contents of fatty acids were measured using an Agilent GC/MS (7890A GC, 240MS) system (Santa Clara, CA, USA).

The specific procedure for determining cell membrane permeability is as follows: Take 5 mL of bacterial culture from each experimental group after incubation, centrifuge at 8000 rpm and 4 °C for 10 min to collect the bacterial cells, wash twice with sterile saline solution (0.85 wt%), and resuspend in an equal volume of saline solution, adjusting the OD600 to 0.5 ± 0.05. Add the fluorescent probe propidium iodide (PI) to a final concentration of 5 μmol/L and incubate in the dark for 30 min at 30 °C, gently inverting and mixing every 5 min. Use a flow cytometer (BD Accuri C6, Franklin Lakes, NJ, USA) to detect fluorescence intensity, with an excitation wavelength of 488 nm and an emission wavelength of 617 nm, analyzing 10,000 cells per sample. Completely lysed bacterial cells (boiled for 10 min) are used as the positive control, and bacterial culture without PI is used as the negative control. Cell membrane permeability is expressed as relative fluorescence intensity (sample fluorescence intensity/positive control fluorescence intensity × 100%).

The steps for extracting and determining cell membrane fatty acids are as follows: Take 10 mL of bacterial culture, centrifuge at 8000 rpm and 4 °C for 10 min to collect the bacterial cells, wash three times with sterile saline to remove residual culture medium, and freeze-dry (−50 °C, 24 h) to constant weight. Add 5 mL of methanol-hydrochloric acid solution (volume ratio 4:1) to the dried bacterial cells and reflux in an 80 °C water bath for 2 h for fatty acid methylation. After cooling to room temperature, add 3 mL of n-hexane and 2 mL of deionized water, vortex for 1 min, and allow the layers to separate before collecting the upper n-hexane phase. The n-hexane phase is dehydrated with anhydrous sodium sulfate, filtered through a 0.22 μm organic phase filter membrane, and analyzed using an Agilent 7890A GC/240MS system. The chromatographic column is an HP-5MS capillary column (30 m × 0.25 mm × 0.25 μm), with the following column temperature program: initial temperature 60 °C (hold for 1 min), increase at 5 °C/min to 250 °C (hold for 10 min). The carrier gas is high-purity helium (flow rate 1.0 mL/min), injection volume 1 μL, split ratio 10:1. The mass spectrometer uses an electron impact ionization source (EI, 70 eV), with a scanning range of *m*/*z* 50–500. Qualitative analysis is performed by searching the NIST 17 mass spectral library and comparing with standards (Sigma-Aldrich, St. Louis, MO, USA). The relative content of each fatty acid is calculated using the peak area normalization method.

### 2.4. DNA Extraction, PCR and High-Throughput Sequencing

Microbial samples were withdrawn from the reactors at the end of operational period. Total genomic DNA of samples was extracted using DNeasy^®^ PowerSoil^®^ Pro Kit (QIAGEN, Duesseldorf, Germany) following the manufacturer’s instructions. The quality of extracted DNA was detected by 1% agarose gel electrophoresis, while the DNA concentration and purity were determined with a NanoDrop 2000 UV-vis spectrophotometer (Thermo Scientific, Wilmington, DE, USA). The Polymerase Chain Reaction (PCR) was performed on the V3-V4 high-variant region fragment of the 16S rRNA gene with primers 338F-806R [57]. The reaction mixture of the PCR contained Forward Primer (5 μM) 0.8 μL, Reverse Primer (5 μM) 0.8 μL, 2× Pro Taq 10 μL, and Template DNA 10 ng/μL, adding ddH_2_O to 20 μL. PCR amplification was conducted using an ABI GeneAmp^®^ 9700 PCR cycler (Applied Biosystems, Foster City, CA, USA) with following program: pre-denaturation at 95 °C for 3 min, followed by 30 cycles including denaturation at 95 °C for 30 s, annealing at 55 °C for 30 s, extension at 72 °C for 45 s, and final extension at 72 °C for 5 min at the end of the cycle. Three replicates were set for each sample. PCR products from the same sample were mixed and detected by 2% agarose electrophoresis, then were purified using the AxyPrep DNA Gel Extraction Kit (Axygen Biosciences, Union City, CA, USA) and quantified using a Quantus™ Fluorometer (Promega, Madison, WI, USA).

Purified amplicons were pooled in equimolar amounts and paired-end sequenced (2 × 300) on an Illumina Miseq platform (Illumina, San Diego, CA, USA) according to the standard protocols by Majorbio Bio-pharm Technology Co., Ltd. (Shanghai, China). The raw sequence data were quality-filtered with Fastp (v.0.19.6) and spliced with Flash (v.1.2.11) to get clean tags. Sequences were assigned to operational taxonomic units (OTUs) at a cutoff of 97% sequence similarity using Uparse (v.11). The sequence number in each sample was rarefied to the same depth, and the taxonomy of each OTU representative sequence was analyzed by RDP Classifier (http://sourceforge.net/projects/rdp-classifier/ (accessed on 22 January 2025)) against the Silva databases (version138/16s_bacteria) using a confidence threshold of 70%.

Analyses of microbial community composition and variations were conducted using the online platform of Majorbio Cloud Platform (https://cloud.majorbio.com (accessed on 22 January 2025)). Alpha diversity indices of the microbial community, including ACE, Chao 1 richness, Shannon and Simpson index, were calculated using Mothur (v.1.30.2). The core microorganism in sulfate-reducing consortium was determined using the heatmap package (v.1.0.8) of R programme (v.3.3.1). Liner Discriminant Analysis (LDA) coupled with Effect Size (LEfSe) was conducted to identify the bacterial taxa differentially represented among sample groupings. The criteria used for the significance level of bacteria at the taxonomic level in different sample groupings was LDA > 3.5 and *p* < 0.05. Networkx (v.1.11) was utilized to construct a species correlation network for the SRB system.

### 2.5. Metagenomic Analysis

Genomic DNA was fragmented to an average size of 400 bp using Covaris M220 (Gene Company Limited, China) for paired-end library construction. The paired-end library was constructed using NEXTflexTM Rapid DNA-Seq (Bioo Scientific, Austin, TX, USA). The sequencing was performed on an Illumina Hiseq Xten platform (Illumina Inc., San Diego, CA, USA) at Majorbio (Shanghai, China), with clean data obtained by trimming any low-quality reads (<50 bp) using Fastp (v. 0.23.0). The redundancy genes were removed and the nonredundant gene catalog was clustered via the Cluster Database at High Identity with Tolerance platform (CD-HIT (v.4.6.1)) at 95% identity and 90% coverage.

The protein sequences translated based on the predicted genes were searched with reference to the KEGG databases (Kyoto Encyclopedia of Genes and Genomes, http://www.genome.jp/kegg/ (accessed on 22 January 2025)) using BLASTP (v.2.2.28+) with an E-value cutoff of 1 × 10^−5^. The genes annotated by KEGG were further assigned to the KEGG ortholog (KO), pathway, EC, and module categories, with the corresponding abundance calculated using SOAPaligner (Version 2.20). Correlation analysis of relative abundance between species and functions was performed using the Python software (v.2.7.0).

### 2.6. Statistical Analysis

Data processing and statistical analysis were performed using Excel 2010 and SPSS 25.0 software (Chicago, IL, USA). The results were presented as mean ± standard deviation (SD) unless stated otherwise. Origin 8.0 was used to create graphics. Significant differences between the experimental groups were identified via one-way analysis of variance (ANOVA) followed by the least significant difference (LSD, *p* < 0.05) test. The Wilcoxon signed-rank test was applied to assess the differences in community alpha diversity. The receiver operating characteristic analysis (ROC) was conducted using the plotROC package of R programme (v.3.3.1). Fisher’s exact test was used to analyze the significance difference in gene abundances.

## 3. Results and Discussion

### 3.1. Effect of pH on Sulfate Reduction Performance

The SRB system exhibited better sulfate reduction performance than the Des. system under acid stress. In general, the pH and SO_4_^2−^ changes in both systems showed a similar trend with decreasing pH (Figure S1). Under neutral conditions, the two systems showed nearly identical treatment performance, with the SO_4_^2−^ removal rates of Des.-CK and SRBs-CK reaching 75.6% and 73.5%, respectively. As the pH decreased from 7.0 to 5.5, the SO_4_^2−^ removal was inhibited, with the sulfate reduction rate of Des.-T1 and SRBs-T1 decreasing by 20.98% and 15.95%, respectively, and sulfate reduction in the former presented a delay of nearly 3 d. Furthermore, as the pH dropped to 5.0, the inhibitory effect of acid stress on sulfate reduction was more pronounced for the Des. system, while the SRB system still achieved sulfate reduction but with a lag of about 7 d, and reached a SO_4_^2−^ removal rate of 30.90%.

### 3.2. Responses of Microbial Growth and Physiological Metabolism to Acid Stress

#### 3.2.1. Responses of Microbial Growth to Acid Stress

The inhibitory effect of acid stress on the growth of microorganisms in the SRB system was less than that in the Des. system. Under neutral conditions, the growth rate of microorganisms was fast in both systems, and OD_600_ reached 0.860 and 0.821 respectively at 7 d (Figure S2a,b), with maximum specific growth rates of 0.23 d^−1^ and 0.10 d^−1^ in Des.-CK and SRBs-CK. As the pH decreased, the growth of microorganisms obviously slowed down; the OD_600_ in Des.-T2 and SRBs-T2 was only 0.43 and 0.65 at the end of the experiments when the pH dropped to 5.0. As shown in Figure S2c, the survival rates of microorganisms in the Des. and SRB systems at pH 5.0 were 5.76% and 31.06%, which were lower than those at pH 5.5 (81.70% and 95.71%, respectively) (*p* < 0.05).

#### 3.2.2. Responses of Physiological Metabolism to Acid Stress

**Figure 1:**
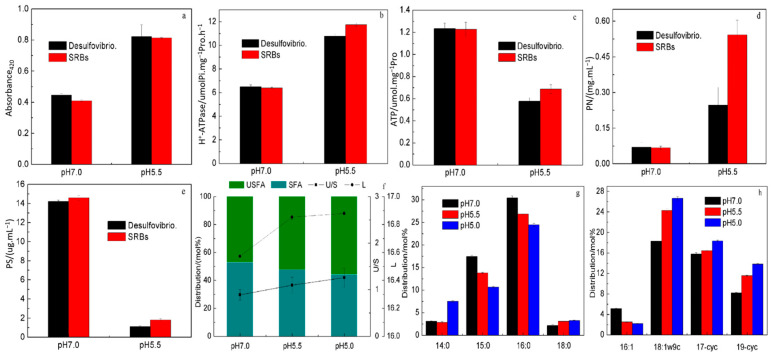
The physiological responses of microorganisms in Des. and SRB systems under different pH conditions (*n* = 3, mean ± SD; different letters indicate significant differences at *p* < 0.05). (**a**) Cell membrane permeability; (**b**) H^+^-ATPase activity; (**c**) Intracellular ATP concentration; (**d**) The content of intracellular protein; (**e**) The content of capsular polysaccharide; (**f**) The unsaturation degree and carbon chain lengths of the cell membrane in SRB system; (**g**) The distribution of saturated fatty acids in SRB system; (**h**) The distribution of unsaturated fatty acids in SRB system.

To elucidate the physiological regulatory mechanisms of microorganisms in response to acid stress, three core indicators were examined: cell membrane integrity, energy metabolism, and intracellular metabolites. Specifically, cell membrane permeability was assessed to reflect membrane structural stability; H^+^-ATPase activity and intracellular ATP concentration were measured to characterize energy metabolism efficiency; and changes in intracellular proteins, capsular polysaccharides, and fatty acid composition were analyzed to reflect adaptive adjustments in metabolites. The results are shown in Figure 1.

As shown in Figure 1a, the cell membrane permeability of cells in Des.-T1 and SRBs-T1 at pH 5.5 was 0.82 and 0.73, respectively, significantly higher than that at pH 7.0 (*p* < 0.05; 0.45 and 0.37 in Des.-CK and SRBs-CK). Figure 1b,c show the changes in H^+^-ATPase activity and intracellular ATP concentration. The H^+^-ATPase activities of cells in Des.-CK and SRBs-CK at pH 7.0 were 6.49 and 6.40 umolPi.mg^−1^pro.h^−1^, respectively, which were lower than those when pH was reduced to 5.5 (10.77 and 11.75 umolPi.mg^−1^pro.h^−1^ in Des.-T1 and SRBs-T1) (Figure 1b). ATP, on the other hand, showed roughly the opposite pattern to H^+^-ATPase. As pH decreased from 7.0 to 5.5, intracellular ATP concentrations in both systems decreased from 1.23 and 1.22 to 0.58 and 0.69, respectively (Figure 1c).

The intracellular protein (PN) contents of the Des. and SRB systems were basically at the same level at pH 7.0, being 0.071 and 0.067, respectively. As the pH decreased, the PN contents in both systems increased to 0.247 and 0.542, and were about 3.48 times and 8.09 times higher than those before the pH drop (Figure 1d). At the same time, it can be seen from Figure 1e that the capsular polysaccharide (PS) contents in Des.-T1 and SRBs-T1 at pH 5.5 were reduced by nearly 13-fold (from 14.2 to 1.11) and 17-fold (from 13.58 to 0.80).

The response of membrane fatty acids to acid stress in the SRB system was further investigated. It is shown in Figure 1f that the content of saturated fatty acids (SFAs) decreased, while the content of unsaturated fatty acids (UFAs) increased with the decrease in pH. The cell membrane unsaturation degree (U/S) at pH 7.0, 5.5, and 5.0 was 0.89, 1.10, and 1.26, respectively, which increased with the acid stress. In addition, the carbon chain length (L) was 16.57, 16.85 and 16.88 at pH 7.0, 5.5 and 5.0, respectively (Figure 1f). The main cell membrane fatty acids detected in the experiment were myristic acid (C14:0), pentadecanoic acid (C15:0), palmitic acid (C16:0), stearic acid (C18:0), palmitoleic acid (C16:1), oleic acid (C18:1ω9c) and cyclopropane fatty acids (C17cyc, C19cyc) (Figure 1g,h). A significantly decrease in C15:0 and C16:0 distribution is shown in Figure 1g, from 17.45 to 13.82 to 10.68 mol% and from 30.44 to 26.84 to 24.46 mol%, respectively. In contrast to C16:1, the levels of other UFAs were elevated under acid stress. The abundances of C18:1ω9c, C17cyc, and C19cyc were progressively higher at lower pH, reaching 24.27%/26.64%, 16.42%/18.34%, and 11.53%/13.81% from baseline values of 18.29%, 15.8%, and 8.20%, respectively (Figure 1h). Furthermore, the results of variance analysis indicated that there were significant differences (*p* < 0.05) in all the detection indicators between the acid stress group (pH 5.5) and the neutral control group (pH 7.0); the SRB system exhibited significantly better acid adaptation ability in terms of H^+^-ATPase activity, intracellular ATP concentration, stress protein synthesis, and adjustment of cell membrane fatty acids compared to the Des. system.

### 3.3. Microbial Community Evolution in SRB System Under Acid Stress

#### 3.3.1. Microbial Community Diversity and Composition

To clarify the impact of acid stress on the microbial community richness and diversity of the SRB system, α-diversity analysis was conducted on the microbial communities in the neutral control group (pH 7.0) and the strong acid stress group (pH 5.0). Core evaluation indices, including the Chao1 index, ACE index (reflecting community richness), Shannon index and Simpson index (reflecting community diversity), were selected for assessment. The results are shown in Table 1. At a similarity level of 97% OTU, the alpha diversity of microbial community in SRB system under pH 7.0 and 5.0 conditions was analyzed. All samples had a coverage rate of >99%, indicating that the amount of sequencing data was large enough to reflect the vast majority of microbial diversity information. The Chao 1 and ACE indices are widely used to represent microbial richness, and the Shannon and Simpson indices can reflect species diversity [58]. As can be seen from Table 1, Chao1, ACE and Shannon indices all decreased slightly under acid stress conditions (SRBs-T2) compared to neutral conditions (SRBs-CK), with no significant difference between treatments (*p* > 0.05).

**Figure 2:**
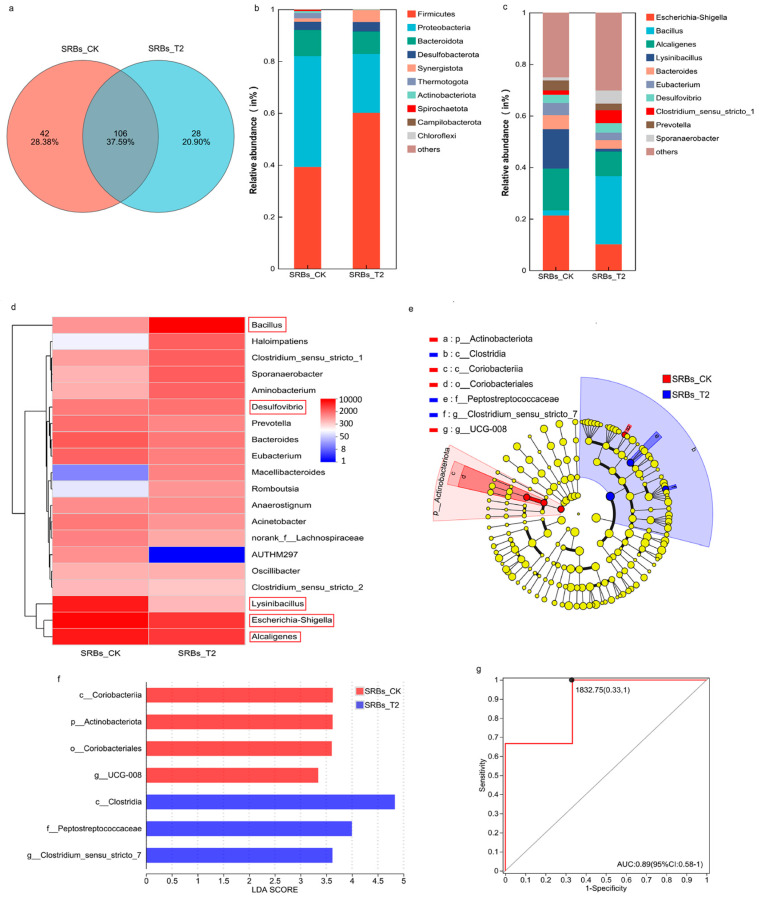
Microbial community in SRBs-CK system (pH 7.0) and SRBs-T2 system (pH 5.0). (**a**) Venn diagram; (**b**) Microbial community structure at the phylum level; (**c**) Microbial community structure at the genus level; (**d**) Clustering heatmap of abundance at the genus level with the 20 highest abundance levels; (**e**) Cladogram showing the phylogenetic distribution of the bacterial lineages; (**f**) LEfSe bar; (**g**) ROC analysis at the genus level.

The effects of acid stress on the microbial community composition, core species, and differential taxa in the SRB system were analyzed through high-throughput sequencing. The OTU Venn diagram illustrates the overlap of community species, the phylum/genus-level structure reveals changes in dominant microbial groups, LEFSe analysis was used to screen significantly different taxa (LDA > 3.0, *p* < 0.05), and ROC analysis validated the diagnostic accuracy of the differential taxa. The detailed results are shown in Figure 2. A total of 176 OTUs were identified after normalization. All of the 16S reads were classified into 12 bacterial phyla, 19 classes, 38 orders and 99 genera. A Venn diagram was used to count the number of OTUs common for the samples in the SRB system at pH 7.0 and pH 5.0, providing a relatively visual representation of the similarity and overlap of OTU composition in the two sets of samples (Figure 2a). There were 106 common OTUs existing in all samples, and the SRBs-CK had 42 unique OTUs while the SRBs-T2 had 28 unique OTUs. The microbial community structures before and after acid stress are shown in Figure 2b,c. Overall, Firmicutes dominated the bacterial communities on the phylum level, representing nearly 50% of the total sequences, followed by Proteobacteria (22.7–42.7%), Bacteroidota (8.7–10.1%), and Desulfobacterota (3.1–3.7%) (Figure 2b). Proteobacteria was the most abundant phylum under neutral conditions (42.7%), followed by Firmicutes (39.2%). However, when the pH dropped to 5.0, the relative abundance of Proteobacteria decreased by over 40%, while Firmicutes increased by 53.3%. At the genus level, the major genera in SRBs-CK included *Escherichia* (21.31%), *Alcaligenes* (16.30%), and *Lysinibacillus* (15.24%). As the pH decreased, the relative abundance of these genera declined in various degrees, while that of *Bacillus* and *Clostridium* increased significantly (reaching 26.26% and 5.14%, respectively) and they became the dominant genera in SRBs-T2 (Figure 2c). The relative abundance of *Desulfovibrio* sp. did not change obviously with the decrease in pH (3.14% before and 3.64% after, respectively).

#### 3.3.2. Changes in Core and Specific Microbes Under Acid Stress

Core microbes were selected based on their relative abundance (>0.01%) and ubiquitousness (prevalence = 1) [59] across the samples in SRB-CK and SRB-T2. A total of 11 bacteria were determined to be core species in the SRB system, accounting for 0.06% of total observed taxa (Table S2). Species within the phyla Proteobacteria, Firmicutes, and Desulfobacterota were predominant taxa. The clustering heatmap of species abundance at the genus levels showed that species within the genus *Bacillus*, *Escherichia-Shigella*, *Alcaligenes*, *Lysinibacillus* and *Desulfovibrio* sp. were predominant taxa (Figure 2d), and the microbial community distribution differed in SRBs-CK and SRBs-T2 under different pH conditions.

In addition, representative differential microbes with significant abundance discrepancies between the SRB-CK and SRB-T2 systems were identified via LEfSe analysis (LDA score > 3.0, *p* < 0.05) (Figure 2e,f). Actinobacteriota was enriched obviously in SRBs-CK, while Firmicutes had a considerable proportion in SRBs-T2. The relative abundance of *Clostridium* in SRBs-T2 was significantly higher than that in SRBs-CK (*p* < 0.05), and ROC analysis further confirmed the high diagnostic accuracy (Figure 2g).

#### 3.3.3. Microbial Community Co-Occurrence Patterns Before and After Acid Stress

**Figure 3:**
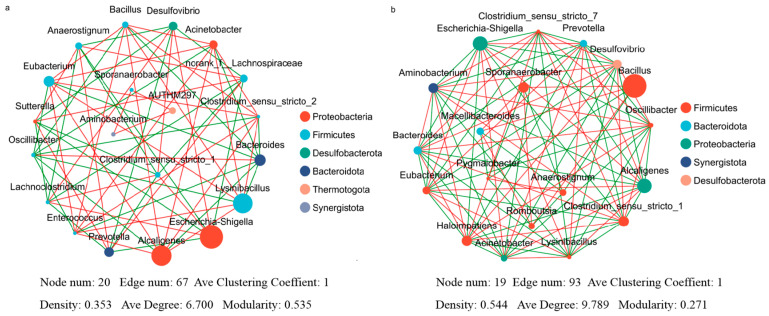
Microbial correlation network at genus level in SRBs-CK system (pH 7.0) (**a**) and SRBs-T2 system (pH 5.0) (**b**). (Connections stand for significant correlations (*p* < 0.05); the size of each node is proportional to the relative abundance of genus; red connecting lines represent positive linear relationships whereas green connecting lines represent negative linear relationships).

The top 20 taxa (at the genus level) in terms of total abundance in the SRB-CK and SRB-T2 systems were selected, and Pearson correlation coefficients among these genera were calculated to characterize interspecific correlations (Figure 3, *p* < 0.05). There was a positive correlation between *Alcaligenes* and *Lysinibacillus*, *Escherichia* and *Desulfovibrio* sp. in SRBs-CK at pH 7.0 (Figure 3a, correlation coefficient > 0). When the pH dropped to 5.0, *Escherichia*, *Alcaligenes* and *Lysinibacillus* in SRBs-T2 were positively correlated with each other and their abundance decreased compared to SRBs-CK (Figure 3b). There was also a positive correlation between *Desulfovibrio* sp. and *Clostridium* sp., which became the dominant genus with an increased abundance, and both were negatively correlated with *Escherichia*, *Alcaligenes*, and *Lysinibacillus* (correlation coefficient < 0). Concurrently, several topological metrics, including network connectivity, network centrality, and modularity, also underwent notable alterations under acid stress.

### 3.4. Functional Characteristics of Metagenomes

#### 3.4.1. Functional Composition of the Sulfate-Reducing Consortium

**Figure 4:**
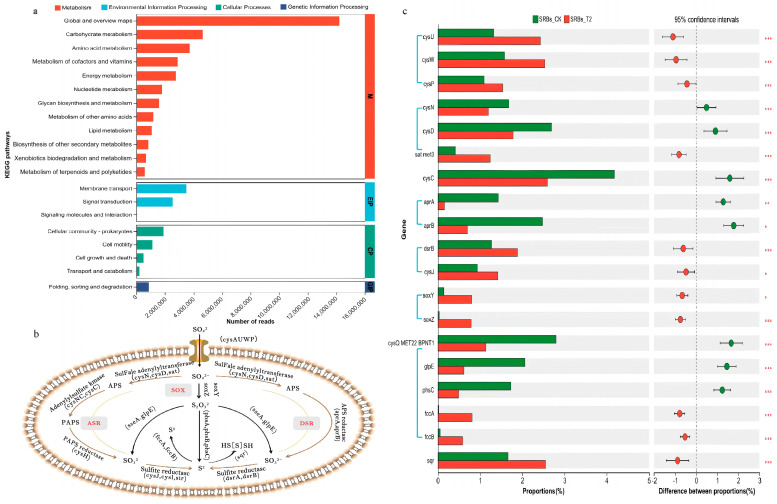
Functional genes of the sulfate-reducing consortium. (**a**) Histogram of KEGG; (**b**) Sulfate-reduction-related pathways; (**c**) The abundance of the top 20 functional genes related to the sulfate reduction in SRBs-CK system (pH 7.0) and SRBs-T2 system (pH 5.0). * *p* < 0.05, ** *p* < 0.01, *** *p* < 0.001.

In total, 6258 KEGG orthologs (KOs) attributed to 313 KEGG pathways were identified from the SRB-CK and SRB-T2 samples (Table S3). Gene functions related to metabolism were abundant accounting for 41.2% of overall annotated sequences (Figure 4a). Specifically, global and overview maps (39.2%), carbohydrate metabolism (12.9%), amino acid metabolism (10.3%), metabolism of cofactors and vitamins (7.9%), and energy metabolism (7.6%) were regarded as the top KEGG functional categories at level 2. At the level of the KEGG pathway, further analysis revealed that starch and sucrose metabolism (ko02010) was the predominant pathway, followed by biosynthesis of cofactors (ko01240), biosynthesis of amino acids (ko01230), two-component system (ko02020), carbon metabolism (ko01200), quorum sensing (ko02024) and purine metabolism (ko00230), complementing the dominant pathways with abundances higher than 3% (Table S4).

#### 3.4.2. Responses of Functional Genes for Sulfate Reduction to Acid Stress

The sulfate metabolic genes in the metagenome gene catalog were annotated using the KEGG database. A total of 73 genes (KEGG orthologs) related to sulfate reduction were detected in the present study (Table S5). These genes were involved in the sulfate import system, assimilatory sulfate reduction (ASR), dissimilar sulfate reduction (DSR), SOX systems, sulfur reduction, sulfur oxidation, sulfur disproportionation, organic sulfur transformation, linkages between inorganic and organic sulfur transformation, and other related metabolism processes [60]. Among them, ASR (M00176), DSR (M00596), and the SOX system (M00595) were three core modules in the sulfate pathway (ko00920).

During the sulfate reduction, cysN, cysD, sat, cysNC, cysC and cysH could biosynthesize an amount of sulfate into sulfur-containing fractions through the ASR pathway, while cysN, cysD, sat, aprA and aprB could reduce more sulfate to sulfides via the DSR pathway to provide energy for the bacteria. cysJ, cysI, sir, dsrA, and dsrB could reduce sulfite to sulfion, while soxY and soxZ could reduce sulfate to thiosulfate (Figure 4b). The change in the abundance of the top 20 functional genes related to sulfate reduction in SRB-CK and SRB-T2 samples was analyzed (Figure 4c). After acid stress, the relative abundance of functional genes including cysC, cysD, cysQ, aprA, aprB, glpE and phsC was significantly lower in SRBs-T2 than in SRB-CK (*p* < 0.001), while the relative abundance of sqr, fccA, fccB, soxY, and soxZ was significantly higher (*p* < 0.001). These changes in gene abundance may reflect potential shifts in functional potential, but do not directly indicate changes in actual functional activity.

### 3.5. The Linking Between the Taxonomic and Functional Properties

**Figure 5:**
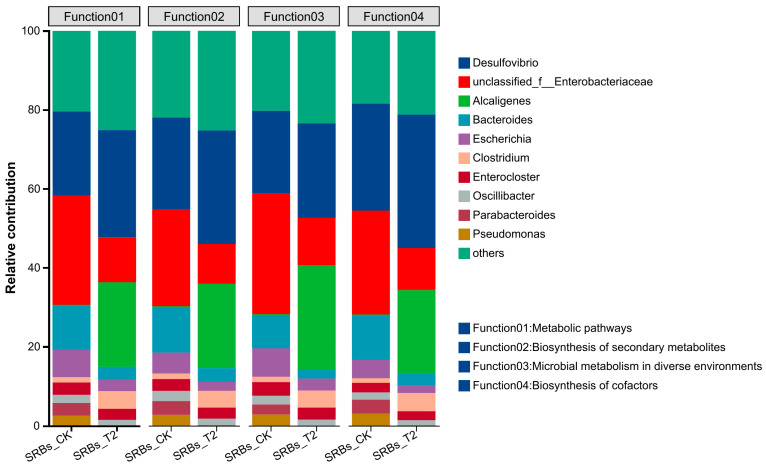
Species and functional contribution in SRBs-CK system (pH 7.0) and SRBs-T2 system (pH 5.0).

To visualize the association between taxonomic and functional properties, the taxonomic origin of functional attributes for the SRB-CK and SRB-T2 samples was determined. The clustered KEGG categories included metabolic pathways, biosynthesis of secondary metabolites, microbial metabolism in diverse environments and biosynthesis of cofactors. As shown in Figure 5, *Desulfovibrio* sp. were the main taxonomic contributor to the above KEGG functional categories, with a relatively higher contribution ratio in SRB-T2 than in SRB-CK. This suggests that *Desulfovibrio* sp. may play a more prominent role in these functions under acid stress, but this inference is based on taxonomic–functional correlation and requires validation by functional protein or metabolite data.

### 3.6. Stability of the Des. and SRB Systems Under Acid Stress

The sulfate reduction performance and microbial growth characteristics of the Des. and SRB systems remained relatively stable and basically at the same level at pH 7.0 according to the SO_4_^2−^ concentration and OD_600_ (Figure S3a). The pH of acid stress-T2 was restored to 7.0, and the changes in SO_4_^2−^ concentration and OD_600_ in both systems are shown in Figure S3b. The resistance (Rs) and resilience (R_L_) of Des.-T2 and SRBs-T2 were calculated (Table S6). The sulfate-reducing ability did not differ much in the recovery phase between the two systems (R_L_ values of 0.205 and 0.22, respectively), but the resistance of sulfate reduction performance in SRBs-T2 to acid stress was 7.57 times higher than that in Des.-T2 (R_S_ values of 0.53 and 0.07, respectively). The Rs values of bacterial density in both systems under acid stress were negative (Table S6), indicating that the decrease in pH had a significant inhibitory effect on microbial growth. Nevertheless, during the pH rebound period, the resilience of the bacterial density in both Des. and SRB systems was strong, with R_L_ of 43.3 and 20.9, and the resilience of Des.-T2 was significantly higher than that of SRBs-T2 (*p* < 0.05).

For the SRB system, the Shannon index and the relative abundance of *Desulfovibrio* sp. were further used as representative indicators to evaluate the resistance and resilience of SRB_S_-T2 in terms of microbial communities under acid stress. The Rs values were 0.97 and 0.73, indicating that the microbial community in the SRB system had a high resistance to pH drop (Table S6). Meanwhile, *Desulfovibrio* sp. had a relative abundance of 56.05% in the recovery period, accounting for an absolute predominance in the microbial community (Figure S3c). The R_L_ value reached 17.85 in the recovery period, showing the strong recovery ability of the SRB system after acid stress.

### 3.7. Effect of Acid Stress on Des. System

Under neutral conditions, the Des. system exhibits excellent sulfate reduction performance with a SO_4_^2−^ reduction rate of 6.91 mg·L^−1^·h^−1^ (Figure S1e). The high sulfate removal efficiency of the Des. system at pH 7.0 is attributed to the neutrophilic characteristics of *Desulfovibrio* sp., whose optimal growth pH ranges from 7.0 to 7.5 [11]. The decrease in pH has adverse effects on the growth and metabolism of *Desulfovibrio* sp. High intracellular and extracellular pH gradients cause diffusion pressure on the cell membrane of microorganisms under acid stress and increase the permeability of the cell membrane (Figure 1a), allowing H+ to freely pass through the cell membrane, which leads to cytoplasmic acidification [61]. In this case, the microorganisms consume most of the energy from the oxidation–reduction process to pump out intracellular protons to maintain pHi homeostasis, and the remaining small amount of ATP will not be enough to supply cell growth and metabolism, thereby affecting the sulfate reduction [7]. It can be seen that *Desulfovibrio* sp. in Des.-T2 entered a dormant state with low growth rate, basically losing the sulfate reduction ability (Figures S1e and S2a).

Nevertheless, the inhibition effect of acid stress (pH 5.0) is highly reversible [62]. In the recovery experiments, *Desulfovibrio* sp. could still multiply rapidly to the level of bacterial density before acid stress (Figure S3b), confirming that the pH drop causes *Desulfovibrio* sp. to be in a sublethal state, but the cells are still active in this state. Therefore, *Desulfovibrio* sp. can adapt to the acidizing environment within a certain range, but once acid stress exceeds the microbial self-regulatory ability, the Des. system then undergoes a prolonged period and cannot guarantee the normal growth of bacteria and the efficient reduction of sulfate, showing limited ability to resist acid stress.

### 3.8. Adaptive Responses to Acid Stress of the SRB System

Compared to the Des. system, the sulfate reduction performance in the SRB system under neutral condition is weaker (Figure S1e), which is related to the microbial community of the system. However, the overall growth rate declines slightly with the extension of reaction time, which is due to the variety of microorganisms in the SRB system. Different bacterial taxa have different growth characteristics, which results in complex interactions such as antagonism or competition during the growth process [54], thereby further affecting sulfate reduction function of the system.

The cell membrane serving as the first barrier is the key site of acid stress, which can regulate the composition and distribution of membrane fatty acids to respond to pH drop, and the literature has shown that there is a certain relationship between the cell membrane fatty acids and bacterial resistance [63]. The main cell membrane fatty acids detected in this study, including C14:0, C15:0, C16:0, C16:1, C18:1w9c, C17cyc and C19cyc, have been reported in many studies on microbial environmental resistance [55,64]. Under acid stress conditions, the fluidity and permeability of the membrane increase [65] (Figure 1a). The above changes in the cell membrane make it easier for extracellular H^+^ to enter the cell and cause disturbance in pH homeostasis, thus seriously affecting the normal physiological state of cell [61]. Therefore, it is crucial to maintain the dynamic balance of intracellular pH, which requires the cell to quickly obtain energy to strengthen the proton-driving force and pump intracellular H^+^ out of the body. Palmgren et al. determined that H^+^-ATPase is a membrane-bound protease with the ability to transport H^+^ across the membrane against the concentration gradient through hydrolyzing intracellular ATP [66]. Similar results have been reported by other scholars in studies on the acid resistance of *Saccharomycetes*, *Bacillus*, etc. [67,68]. Moreover, the decrease in pH may affect the production of cellular metabolites [69]. But omics exploration is needed in the future to determine which type of proteins are involved. At the same time, most of the energy has been consumed in the process of pumping out intracellular H^+^, suggesting that reducing the secretion of PS in response to acidification is also a strategy to improve the acid adaptation of the SRB system [70].

Studies have shown that [71] CFA, as a basic component of the cell membrane, plays an important protective role in the biophysical properties of the lipid bilayer as well as in the growth and survival of microorganisms, which can be synthesized to accelerate by microorganisms under harsh conditions [72]. Jiang et al. studied the physiological characteristics of Helicobacter pylori in the human gastrointestinal environment and found that CFA modification of membrane lipids is vital for bacteria to adapt to pH changes [53]. In fact, high proportions of CFA (5–30%) in the cell membranes have been detected in studies on acid resistance of some bacteria (*Escherichia coli*, *Lactobacillus casei*, *Salmonella typhimurium*, etc.) [73,74], and similar protective effects of CFA have also been confirmed in bacteria under other environmental stresses such as temperature, humidity, salinity and osmotic pressure [55,75]. However, it is worth noting that the formation of cyclopropane rings requires the consumption of three ATP molecules [53], which is an energy-expensive modification process.

**Figure 6:**
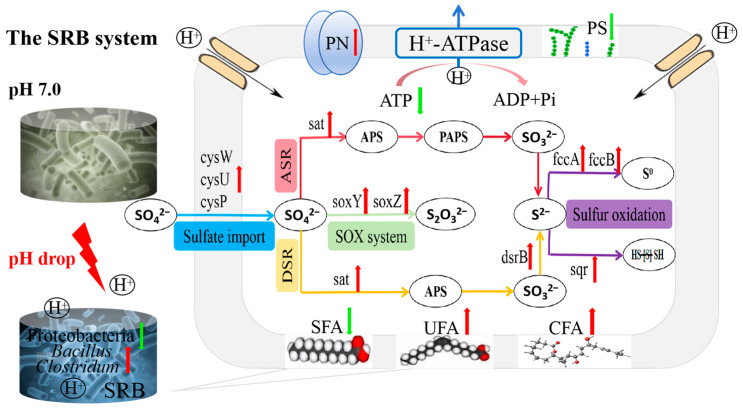
The acid-adaptive mechanisms of the sulfate-reducing consortium in SRB system. (Fine arrows of different colors represent different sulfate-reduction-related pathways. The blue fine arrows represent sulfate import; the yellow fine arrows represent dissimilar sulfate reduction; the pink fine arrows represent assimilatory sulfate reduction; the purple fine arrows represent sulfur oxidation; the light-green fine arrows represent the SOX system. The red upward thick arrows indicate an increase in content or abundance, whereas the green downward thick arrows indicate a decrease in content or abundance).

Studies have shown that *Bacillus* has sufficient adaptability to acidic environments [76], responding to acid stress effectively by increasing the activity of glutathione peroxidase (GPX) and the content of oxidized glutathione (GSSG) [77]. As for *Clostridium*, a large number of studies have reported that it may be more tolerant to various environmental pressures such as acidity, alkalinity, salinity, etc. [20,78,79] than other species because of its predominance in SRB communities [80,81], and it is considered as a suitable biocatalyst for treating sulfate-rich wastewater. According to the microbial correlation network (Figure 3), *Bacillus*, *Clostridium* and *Desulfovibrio* sp. coexist with high abundance in the SRB system under acid stress, indicating that there may be a cooperative relationship between them, which is helpful for improving the acid adaption, thus providing a favorable environment for *Desulfovibrio* sp. to reduce sulfate. These synergistic symbiotic microorganisms acquire unique ecological niches in the system, thereby jointly enhancing the acid resistance and sulfate reduction performance of the SRB system (Figure 3b) [82]. But the decrease in pH alters the proportional distribution of functional genes related to sulfate reduction in the SRB system [41,83], which promotes the sulfate reduction overall. In summary, the underlying acid-adaptive mechanisms of the SRB system under acid stress are shown in Figure 6.

## 4. Conclusions

This study systematically elucidated the efficient adaptation mechanisms and sulfate removal advantages of sulfate-reducing bacterial communities (the SRB system) under acid stress by integrating physiological, ecological, and molecular approaches, thereby addressing the overall knowledge gap in previous research. Comparative experiments revealed that as pH decreased, systems inoculated solely with *Desulfovibrio* exhibited significant inhibition in microbial growth and sulfate reduction, whereas the SRB system demonstrated strong adaptive capacity and maintained excellent performance. From a physiological perspective, the study quantified key indicators such as H^+^-ATPase activity, cyclopropane fatty acid content, and stress protein production, clarifying that the SRB system enhances acid tolerance by regulating membrane integrity, energy metabolism, and intracellular metabolite synthesis. From a community perspective, high-throughput sequencing identified an acid-tolerant synergistic network dominated by *Bacillus*, *Clostridium*, and *Desulfovibrio*, with co-occurrence analysis further elucidating their cooperative coexistence patterns. At the functional gene and metabolic network levels, metagenomic analysis indicated that the differential expression of core functional genes (e.g., *cysW*/*cysP*, *dsrB*, *soxY*/*soxZ*) is directly related to shifts in taxonomic structure, thereby establishing a link between microbial community evolution and enhanced metabolic functions in acidic environments. This multi-scale research framework overcomes the limitations of previous studies that relied on single indicators, constructing a comprehensive adaptation pathway for the SRB system ranging from physiological regulation and population cooperation to gene expression, thus providing a crucial theoretical foundation for its practical application in treating sulfate-containing acidic wastewater. However, this work has several limitations that require further investigation: First, batch experiments only reflect short-term acid stress responses and fail to reveal the long-term (months to years) evolutionary patterns of microbial communities and cumulative gene mutations under acid acclimation. Additionally, the dynamic fluctuations of pH in actual wastewater and their impact on the long-term stability of the system were not considered, necessitating validation through continuous-flow reactors or pilot-scale experiments. Second, the experiments used a simplified, single-component culture medium, which did not account for complex factors present in actual wastewater (e.g., high concentrations of heavy metal ions, coexisting pollutants, and high salinity in acid mine drainage). These factors may exhibit synergistic toxicity with H^+^ or alter metabolic pathways, requiring further optimization of the conclusions for practical applications. Thirdly, although a dominant symbiotic system was identified, the specific synergistic mechanisms among *Bacillus*, *Clostridium*, and *Desulfovibrio* (such as metabolite exchange and gene co-expression) and their functional roles in sulfate reduction (direct involvement or indirect assistance) remain to be thoroughly elucidated. Lastly, current data on gene expression and functional protein validation are insufficient. The available data only pertain to the gene level, lacking evidence from transcriptomics and proteomics. Overall, this study clarifies the stress responses and intrinsic acid adaptation mechanisms of sulfate-reducing consortia, providing critical theoretical insights for their practical application in the treatment of acidic sulfate-containing wastewater.

## Figures and Tables

**Table 1:** α-Diversity indices of the SRB system microbial community under neutral conditions (SRBs-CK, pH 7.0) and strongly acidic conditions (SRBs-T2, pH 5.0) (similarity level 97%). (*n* = 3, mean ± SD).

Treats	Similarity Level: 0.97				
	Coverage	Chao1 Index	ACE Index	Simpson Index	Shannon Index
SRBs-CK	0.999	93.56	97.91	0.16	2.52
SRBs-T2	0.999	92.54	93.66	0.16	2.48

## Data Availability

The original contributions presented in this study are included in the article/Supplementary Materials. Further inquiries can be directed to the corresponding author.

## Supplementary Materials

The following supporting information can be downloaded at https://www.mdpi.com/article/10.3390/biom16030444/s1. Text S1. Assessment for the Recoverability of The Des. and SRBs Systems. Text S2. The Percent Cell Survival Measurements. Text S3. The Membrane Permeability Measurements. Text S4. Extraction of Fatty Acids in Cell Membrane and GC/MS Analysis. Figure S1. The sulfate reduction of the Des. and SRBs systems under different pH conditions. (a) pH of the Des. system effluent. (b) pH of the SRBs system effluent. (c) SO_4_^2−^ concentration of the Des. system effluent. (d) SO_4_^2−^ concentration of the SRBs system effluent. (e) The SO_4_^2−^ removal rate and sulfate reduction rate. Figure S2. The growth of microorganisms in Des. and SRBs systems under different pH conditions. (a) Microbial growth curve in the Des. system. (b) Microbial growth curve in the SRBs system. (c) The percent cell survival. Figure S3. Stability of the Des. and SRBs systems. (a) SO_4_^2−^ concentration and OD600 in Des.-CK and SRBs-CK systems (pH 7.0); (b) SO_4_^2−^ concentration and OD600 in Des.-T2 and SRBs-T2 systems (pH 5.0); (c) Microbial community structure in the SRBs system at steady-state inhibition stage (SRBs-T2) and recovery stage (SRBs-T2-R). Table S1. The experimental groups of the pH influencing study. Table S2. The OTU abundance. Table S3. The KO abundance-Bacteria. Table S4. KEGG-pathway. Table S5. The KO abundance-ko00920. Table S6. The resistance (RS) and resilience (RL) of the Des.-T2 and SRBs-T2 systems.
