# Supplementary material for: Effects and Adaptive Responses of Sulfate-Reducing Biochemical System to Acid Stress
Source: Biomolecules. 2026 Mar 16;16(3):444. doi: 10.3390/biom16030444 (PMC13023848; doi:10.3390/biom16030444)
Supplement: Supplementary file 1 [file biomolecules-16-00444-s001.zip › biomolecules-4115905-supplementary.pdf]

Supplementary Information

Effects and adaptive responses of sulfate-reducing biochemical system to acid stress

This PDF file includes:

Number of Pages: 4

Supporting Texts S1-S4

Tables S1-S6

Figures S1-S3

Supplementary Methods

Text S1. Assessment for the Recoverability of The Des. and SRBs Systems.

Taking the control groups under neutral condition (Des.-CK and SRBs-CK) as the reference states, the sulfate reduction efficiency and bacterial density were used as the system functional response indicators, while the alpha diversity and the abundance of key species as the microbial community response indicators. The resistance and resilience of the Des. and SRBs systems to acid stress treatments (Des.-T2 and SRBs-T2) were calculated based on the following formula:

$$R_S = 1 - \left(\frac{2\left|C_0 - P_0\right|}{C_0 + \left|C_0 - P_0\right|}\right) \quad (1)$$

$$R_L = \frac{P_x}{C_0} \quad (2)$$

where R_S represents the resistance, R_L represents the resilience. Using the sulfate reduction efficiency as a one-dimensional response index to calculate the stability of

T2 as an example, C_0 refers to the initial sulfate concentration in the system during the stress test, P_0 refers to the sulfate concentration in the system at the end of the acid stress treatment, and Px refers to the sulfate concentration in the system at the end of the recovery test. Other indicators can be calculated in a similar manner.

Text S2. The Percent Cell Survival Measurements.

The dilution plate counting method was used for the percent cell survival. The cell suspensions were serially diluted and plated on Columbia blood plate for CFU counts after 15 days of incubation under an anaerobic growth condition. The percent cell survival at pH 5.5 or pH 5.0 relative to that at pH 7.0 was calculated.

Text S3. The Membrane Permeability Measurements.

Cells of logarithmic growth phase cultured in different treats were collected by centrifugation (6，000×g, 4℃, 10 min) and washed twice with phosphate buffer solution (10mM, pH7.4) followed by resuspending in the same buffer to adjust the cell density to appropriate concentration (OD600=1.0). A certain amount of 2-Nitrophenyl β-D-galactopyranoside was added to 1mL of just mentioned bacterial suspension and the final concentration was adjusted to 100 μg/mL. The absorbance at 420 nm was measured by spectrophotometer, which was used to express the membrane permeability.

Text S4. Extraction of Fatty Acids in Cell Membrane and GC/MS Analysis.

Cells of logarithmic growth phase cultured in different treats were collected by centrifugation (8, 000×g, 4℃, 10 min) and washed twice with sterilized deionized water followed by discarding supernatants. The pellet was placed in a water bath at

70°C for 30 min after adding 2 mL of sodium methoxide (1 M) and shaked every 10 minutes during the process, then it was allowed to cool at room temperature. The residual matter was mixed with 4 mL methyl ester solution vibrately and heated at 80°C for 30 min. Then the 1.25 mL of chromatographic grade n-hexane and 2 ml of 3% saturated NaCl was added successively, and the test tube was tumbled for 5 min. Subsequently, the solution was layered after centrifuging at 10, 000×g, 4℃ for 3 min. The content of the fatty acid methyl esters (FAMEs) in the upper layer of the organic phase was measured using an Agilent GC/MS (7890A GC, 240MS) system. The degree of unsaturation (U / S), the certain fatty acid distribution (mol %) and the mean chain length (L) were calculated as:

$$U / S = UFAs / SFAs \quad (3)$$

Distribution=certain fatty acid peak area/total fatty acid peak×100% （4）

$$L = \frac{\sum C \times FA}{\sum FA} \quad (5)$$

where FA represents the fatty acid, UFA denotes the the content of unsaturated fatty acid, SFA denotes the content of saturated fatty acid, C denotes the number of carbon atoms in the fatty acid.

Supplementary Tables

Table S1. The experimental groups of the pH influencing study.

Treats	Des. system			SRBs system		
	Des.-CK	Des.-T1	Des.-T2	SRBs-CK	SRBs-T1	SRBs-T2
pH	7.0±0.1	5.5±0.1	5.0±0.1	7.0±0.1	5.5±0.1	5.0±0.1

Note: The system inoculated with *Desulfovibrio* in this study was denoted as the Des. system, while the system inoculated with the sulfate-reducing consortium was recorded as the SRBs system.

Table S2. The OTU abundance.

OTU	Phylum	Genus	Total Abundance	Relative Abundance (%)	Prevalence (%)
OTU139	Proteobacteria	Escherichia-Shigella	37956	15.7	100
OTU157	Firmicutes	Bacillus	34365	14.2	100
OTU145	Proteobacteria	Alcaligenes	31146	12.9	100
OTU179	Firmicutes	Lysinibacillus	19560	8.1	83.3
OTU180	Firmicutes	Eubacterium	8948	3.7	100
OTU130	Bacteroidota	Prevotella	7711	3.2	66.7
OTU135	Firmicutes	Sporanaerobacter	7612	3.1	50.0
OTU64	Synergistota	Aminobacterium	7145	3.0	50.0
OTU160	Proteobacteria	Acinetobacter	6293	2.6	83.3

OTU141	Firmicutes	Haloimpatiens	6269	2.6	83.3

Table S3. The KO abundance-Bacteria.

KO	KEGG Name	Total Reads	Gene Count	Pathway	KO Description
K03406	mcp	237,934	723	ko02020;ko02030	methyl-accepting chemotaxis protein
K07497	K07497	209,156	461	NA	putative transposase
K21572	susD	160,904	1057	NA	starch-binding outer membrane protein
K21636	nrdD	134,062	169	ko00240;ko00230	ribonucleoside-triphosphate reductase
K02004	ABC.CD.P	109,270	615	NA	putative ABC transport system permease protein
K07090	K07090	102,364	174	NA	uncharacterized protein
K03465	thyX, thy1	92,268	38	ko00240;ko01232	thymidylate synthase (FAD)
K04068	nrdG	90,990	67	NA	anaerobic ribonucleoside-triphosphate reductase

K01999	livK	86,350	161	ko02024;ko02010	branched-chain amino acid transport system
K02529	lacI, galR	73,128	288	NA	LacI family transcriptional regulator
K01995	livG	69,706	120	ko02010;ko02024	branched-chain amino acid transport system
K07486	K07486	65,688	133	NA	transposase
K17680	PEO1	65,306	21	ko05017	twinkle protein
K00986	ltrA	63,358	183	NA	RNA-directed DNA polymerase
K01998	livM	62,444	120	ko02010;ko02024	branched-chain amino acid transport system

Table S4. KEGG-pathway.

Pathway	Pathway Name	Group A4 (Abundance)	Group C (Abundance)	Fold Change (A4/C)	Abundance Difference
ko01100	Metabolic pathways	0.1991	0.1828	1.09	0.0163
ko01110	Biosynthesis of secondary...	0.0825	0.077	1.07	0.0055
ko01120	Microbial	0.0555	0.0527	1.05	0.0027

	metabolism in...				
ko02010	ABC transporters	0.0353	0.0393	0.9	-0.0040
ko01240	Biosynthesis of cofactors	0.0345	0.033	1.05	0.0015
ko01230	Biosynthesis of amino acids...	0.0338	0.0321	1.05	0.0017
ko02020	Two-componen t system	0.0331	0.0289	1.15	0.0042
ko01200	Carbon metabolism	0.0293	0.0275	1.07	0.0018
ko02024	Quorum sensing	0.0148	0.0178	0.83	-0.0030
ko00230	Purine metabolism	0.0167	0.0157	1.07	0.001
ko01232	Nucleotide metabolism	0.013	0.0137	0.95	-0.0006
ko00620	Pyruvate metabolism	0.0137	0.0124	1.11	0.0013

Table S5. The KO abundance-ko00920.

Pathway	Pathway Name	A4 Sample	C Sample	Total

		Abundance	abundance	abundance
ko01200	Global and overview maps	0.201568	0.18038	0.381948
ko02010	ABC transporters	0.068123	0.056444	0.124567
ko00195	Photosynthesis	0.042156	0.038972	0.081128
ko00900	Terpenoid backbone biosynthesis	0.038765	0.035219	0.073984
ko02020	Two-component system	0.014366	0.016892	0.031258
ko00500	Starch and sucrose metabolism	0.015872	0.014985	0.030857
ko00220	Arginine biosynthesis	0.015621	0.014783	0.030404
ko00230	Purine metabolism	0.015236	0.014587	0.029823
ko00240	Pyrimidine metabolism	0.014892	0.014235	0.029127
ko00190	Oxidative phosphorylation	0.014658	0.013982	0.02864
ko00010	Glycolysis / Gluconeogenesis	0.012419	0.010984	0.023403
ko00999	Biosynthesis of various secondary	0.011258	0.010309	0.021567
ko00020	Citrate cycle (TCA cycle)	0.007529	0.006792	0.014321
ko00030	Pentose phosphate pathway	0.007175	0.005808	0.012982
ko00051	Fructose and mannose metabolism	0.00733	0.005091	0.012421

Table S6. The resistance (Rs) and resilience (R_L) of the Des.-T2 and SRBs-T2 systems.

Stability of systems		Sulfate reduction（SO_4^{2-} concentration）	Bacterial density（OD_{600}）	Alpha diversity（Shannon index）	The abundance of key species（*Desulfovibrio*）
Des.-T2	Rs	0.07	-0.91	-	-
	R_L	0.205	43.3	-	-
SRBs-T2	Rs	0.53	-0.88	0.97	0.73
	R_L	0.22	20.9	0.60	17.85

Supplementary Figures

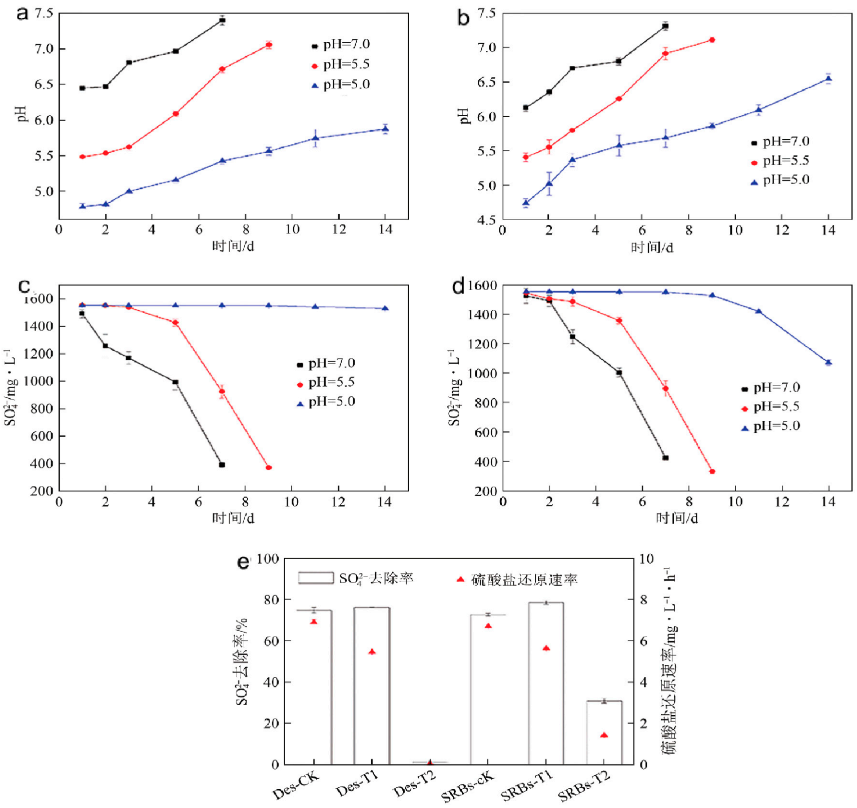

Figure S1. The sulfate reduction of the Des. and SRBs systems under different pH conditions. (a) pH of the Des. system effluent. (b) pH of the SRBs system effluent. (c) SO_4^{2-} concentration of the Des. system effluent. (d) SO_4^{2-} concentration of the SRBs system effluent. (e) The SO_4^{2-} removal rate and sulfate reduction rate.

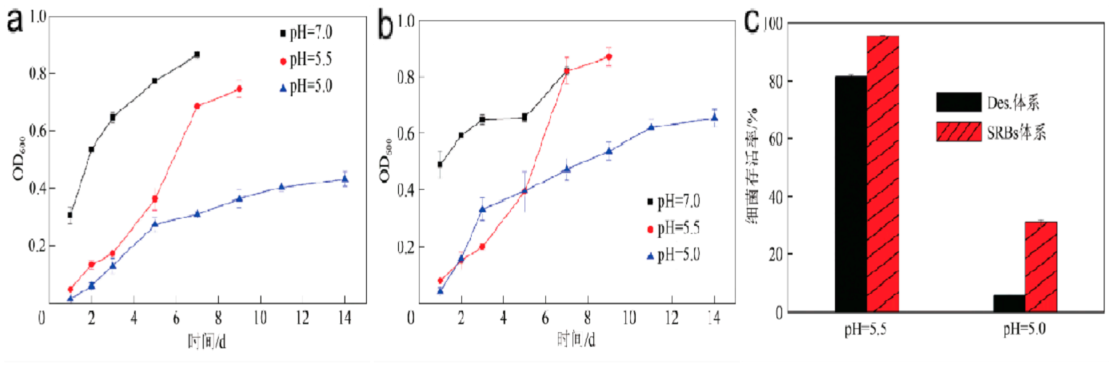

Figure S2. The growth of microorganisms in Des. and SRBs systems under different pH conditions. (a) Microbial growth curve in the Des. system. (b) Microbial growth curve in the SRBs system. (c) The percent cell survival.

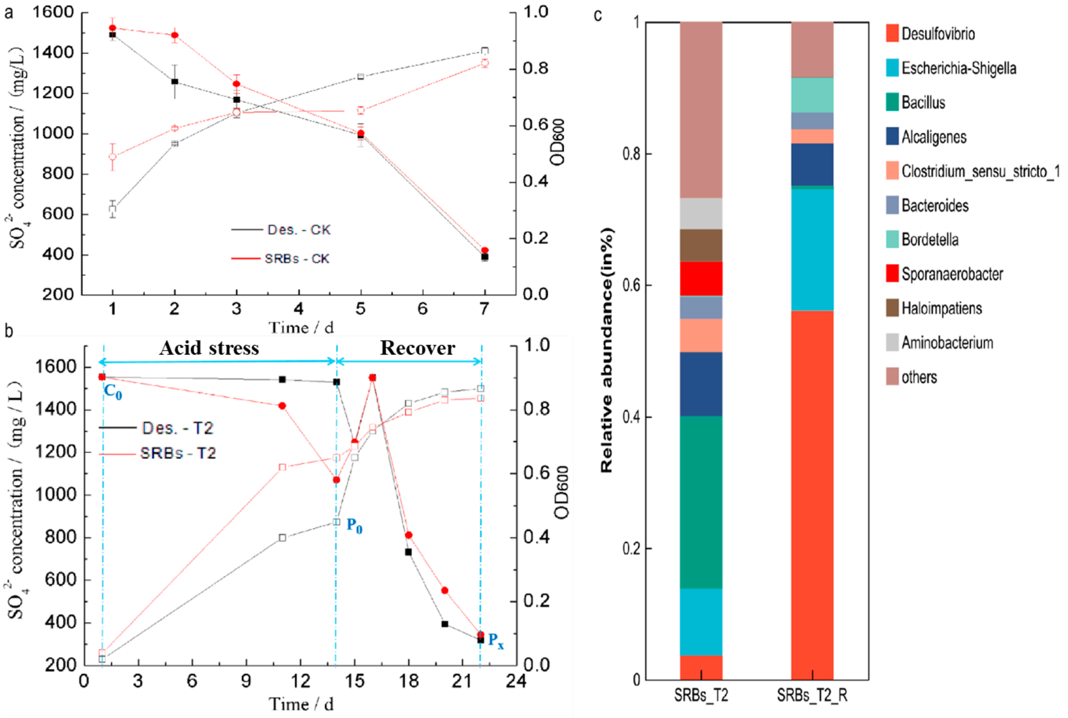

Figure S3. Stability of the Des. and SRBs systems. (a) SO_4^{2-} concentration and OD_{600} in Des.-CK and SRBs-CK systems (pH 7.0); (b) SO_4^{2-} concentration and OD_{600} in Des.-T2 and SRBs-T2 systems (pH 5.0); (c) Microbial community structure in the SRBs system at steady-state inhibition stage (SRBs-T2) and recovery stage (SRBs-T2-R).
